# VOLUNTEERING AMONG ECONOMICALLY MARGINALIZED OLDER ADULTS IN THE US: A SCOPING REVIEW

**DOI:** 10.1093/geroni/igae098.1854

**Published:** 2024-12-31

**Authors:** Byeongju Ryu, Christina Matz, Cal Halvorsen, Jihye Baek

**Affiliations:** Washington University in St. Louis, St. Louis, Missouri, United States; Boston College, Chestnut Hill, Massachusetts, United States; Washington University in St. Louis, St. Louis, Missouri, United States; Washington University in St. Louis, St. Louis, Missouri, United States

## Abstract

The 2015 White House Conference on Aging highlighted civic and volunteer activities for healthy aging. While the benefits of volunteering in later life are well-documented, the experiences of economically marginalized older adults are less understood. This study systematically reviewed evidence on volunteering among this group in the U.S., focusing on their factors and outcomes. The study reviewed empirical research published since 2015, obtained from five electronic databases, selecting 11 studies focusing on older volunteers with low income or assets. The selection was based on 1) Samples predominantly aged 50+ or defined as older adults and 2) A focus on volunteering among low-income or asset older people. Reported facilitators included personal values, perceived technology utility and accessibility, linguistic and cultural adaptations in program designs, skill acquisition opportunities, post-retirement work, and living in senior housing. Barriers encompassed low technological and English proficiency, limited resources, and social distancing policies. Outcomes involved enhanced purpose and engagement in life, skill development, expanded social networks and community connectedness, improved self-reported health, and fewer depressive symptoms. This scoping review focuses on an under-researched group: economically marginalized older volunteers—underscoring the need to encourage their volunteerism. Volunteering could improve their health and well-being, potentially reducing dependence on social insurance, boosting the gross domestic product, and strengthening the nonprofit sector. Given the primary focus on formal volunteering and cross-sectional study designs in the reviewed literature, future investigations may examine informal volunteering and utilize longitudinal or experimental methodologies to examine the causal relationships of their volunteering with factors and outcomes.

